# *Notes from the Field:* Multistate Outbreak of *Escherichia coli* O157:H7 Infections Linked to a National Fast-Food Chain — United States, 2022

**DOI:** 10.15585/mmwr.mm7226a6

**Published:** 2023-06-30

**Authors:** Christan Stager, Danielle Donovan, Lauren Edwards, Evelyn Pereira, Laurie Williams, Jennifer Freiman, Colin Schwensohn, Laura Gieraltowski

**Affiliations:** ^1^Epidemic Intelligence Service, CDC; ^2^Michigan Department of Health and Human Services; ^3^Michigan Department of Agriculture and Rural Development; ^4^Coordinated Outbreak Response and Evaluation Network, Food and Drug Administration, College Park, Maryland; ^5^Retail Food Policy Team, Office for Food Safety, Center for Food Safety and Applied Nutrition, Food and Drug Administration, Silver Spring, Maryland; ^6^Office of Public Health Science, Food Safety and Inspection Service, U.S. Department of Agriculture, Washington, DC; ^7^Division of Foodborne, Waterborne, and Environmental Diseases, National Center for Emerging and Zoonotic Infectious Diseases, CDC.

In August 2022, the Michigan Department of Health and Human Services alerted CDC to an approximately fivefold increase in regional cases of *Escherichia coli* O157:H7 infection. Whole genome sequencing was used to characterize isolates from laboratory-confirmed infections in ill persons. Initial patient interviews indicated that many had consumed meals from the same national fast-food chain. Federal, state, and local officials initiated an investigation to identify the outbreak source and prevent additional cases. This activity was reviewed by CDC and was conducted consistent with applicable federal law and CDC policy.*

CDC defined a case as an *E. coli* O157:H7 infection with an isolate highly related to the outbreak strain (within 0–2 alleles) by core genome multilocus sequence typing, with illness onset during July 26–August 24, 2022. PulseNet, CDC’s national molecular subtyping network for enteric disease surveillance, detected 109 cases from six states, including Michigan (67; 61%), Ohio (24; 22%), Indiana (11; 10%), Pennsylvania (four; 4%), Kentucky (two; 2%) and New York (one; 1%). The median patient age was 22 years (range = 1–94 years), and 49 (45%) were female. Fifty-two (48%) patients were hospitalized, and 13 (12%) developed hemolytic-uremic syndrome, a recognized complication of *E. coli* O157:H7 infection; no deaths occurred.

Hypothesis-generating interviews were conducted with 84 (77%) patients; among these, 70 (83%) reported eating at the same fast-food chain during the week preceding illness onset. Investigation identified 11 restaurant clusters (groups of unrelated ill persons who ate at the same restaurant). Ill persons reported eating food ingredients commonly served together on several menu items. Among 68 patients who provided detailed information, the most commonly reported exposures were beef patties (53; 78%) and romaine lettuce on sandwiches (46; 68%). Early in the investigation, romaine lettuce exposure exceeded 90%, prompting the fast-food chain to remove lettuce in states with outbreak-associated cases. Food handlers infected with the outbreak strain were identified but were unlikely to be the ultimate source. Although ill food handlers might have amplified the outbreak at some locations, many restaurant clusters had no affected food handlers. 

Considering menu items reported by ill persons, and that foodborne *E. coli* O157:H7 outbreaks are often linked to leafy greens and beef (*1*), the Food and Drug Administration (FDA) traced romaine lettuce and the U.S. Department of Agriculture’s Food Safety and Inspection Service (USDA-FSIS) traced beef patties to determine their source. Neither traceback identified a single production lot that could explain all outbreak-associated illnesses. In the absence of another restaurant cluster outside of the national fast-food chain, FDA and USDA were unable to use triangulation to identify convergence of a specific food item to a common source. States tested food from restaurants, and FDA tested foods and environmental samples from the supply chain; however, the outbreak strain was not identified in the tested samples.

Investigators linked this large multistate outbreak of *E. coli* O157:H7 infections to eating at a national fast-food chain. Despite epidemiologic, traceback, and microbiologic investigations, the contaminated ingredient was not confirmed. This outbreak highlights recurring challenges associated with investigating outbreaks linked to single restaurant chains (*2*,*3*). Ingredient collinearity (i.e., the sharing of many ingredients among multiple menu items) precluded identification of a single item associated with illnesses. Cross-contamination among ingredients or from ill food handlers also complicated source identification. The absence of restaurant clusters with an independent supply system outside the fast-food chain prevented use of triangulation to identify the source (*4*). Despite these challenges, clear communication with state partners, FDA, USDA-FSIS, and the restaurant chain led to rapid public health action to remove suspected romaine lettuce from identified restaurants. No outbreak-associated illnesses were reported after the suspected romaine lettuce was removed.

## Footnotes

* 45 C.F.R. part 46.102(l)(2), 21 C.F.R. part 56; 42 U.S.C. Sect. 241(d); 5 U.S.C. Sect. 552a; 44 U.S.C. Sect. 3501 et seq.
